# Aspirin use and pancreatic cancer risk: A systematic review of observational studies

**DOI:** 10.1097/MD.0000000000018033

**Published:** 2019-12-20

**Authors:** Jinjin Sun, Yanxun Li, Lili Liu, Zhijia Jiang, Geng Liu

**Affiliations:** a Department of Hepatopancreatobiliary Surgery, The Second Hospital of Tianjin Medical University, Tianjin Medical University; b Pediatric Surgery, Tianjin Children's Hospital, Tianjin, China.

**Keywords:** aspirin, meta-analysis, pancreatic cancer, risk

## Abstract

**Objectives:**

Although there is evidence that aspirin might be able to prevent pancreatic cancer, the findings have been inconsistent. In this paper, we conducted a meta-analysis of observational studies to examine the relationship between aspirin use and the risk of pancreatic cancer.

**Methods:**

We identified potential studies by searching the MEDLINE, EMBASE, and Wangfang (Chinese database) database (from 1967 to March 2017) and by reviewing the bibliography of relevant publications. Random effects model was used to calculate odds ratio (OR) and 95% confidence interval. The Cochran Q statistic (significance level at *P* < .1) was used to assess heterogeneity in this study. The author adopted weighted regression method of Egger to assessed publication bias.

**Results:**

A total of 12 studies involving 4748 pancreatic cancer cases, were included in the meta-analysis. The study reflected that there was no signification association between aspirin use and mortality risk of pancreatic cancer. Aspirin use might reduce the incidence of pancreatic cancer. Specifically, there was a high signification association between frequent aspirin use and reduced pancreatic cancer incidence, without heterogeneity. In addition, there was a high signification association between duration of aspirin use more than 5 years and reduced pancreatic cancer incidence, without obvious heterogeneity among the original studies.

**Conclusions:**

In summary, this meta-analysis suggested that the aspirin use might be negatively related to the incidence risk of pancreatic cancer. Specifically, the frequency and duration of aspirin use might play an important role in decreasing the incidence of pancreatic cancer.

## Abstract 2

Supplemental Digital Content is available in the text

## 1 Introduction

Pancreatic cancer is one of the most common gastrointestinal tumors and is becoming one of the main causes of cancer related deaths in the world.^[1]^ Difficulties in early diagnosis, local invasion, and rapid progression are the characteristics of pancreatic cancer, which has a high degree of malignant disease and a mortality almost equal to its incidence.^[1,2]^ A Cancer Statistics Report in 2017 for Americans showed that there were 53,670 new cases of pancreatic cancer diagnosed and an incidence of 43,090 deaths. The incidence of pancreatic cancer is fourth of the most common types of malignant tumors.^[2]^ Therefore, it is highly urgent to identify potential chemoprevention agents for pancreatic cancer.

Aspirin, a classic and remarkable drug in the non-steroidal anti-inflammatory drugs (NSAIDs) family, has been extensively researched in cardiovascular disease and has been widely used for the treatment of pain, fever, and other inflammatory conditions for more than a century.^[3]^ Aspirin shows major pharmacological effects by inhibiting both COX-1 and COX-2.^[4]^ Large-scale studies have showed that the overproduction of COX-2 in pancreatic lesions is similar to many other malignant tissues.^[5,6]^ Inhibition of cyclooxygenase (COX) enzymes by NSAIDs might inhibit cell proliferation and tumor angiogenesis.^[7,8]^ As a potential anti-tumor agent, aspirin has been widely studied for chemoprevention of various cancers, including colorectal, breast, prostate, esophageal, gastric, lung, ovarian, and pancreatic cancer.^[9–19]^ The laboratory studies have demonstrated that inhibition of COX-2 activity might be an effective preventive method for the incidence of pancreatic cancer.^[20,21]^

However, it remains controversial between aspirin use and risk of pancreatic cancer. There are several studies on the above controversial issues. Larsson et al^[22]^ found no association between aspirin use and pancreatic cancer risk. In 2007, Capurso et al^[23]^ concluded that there was no relationship between the use of aspirin and pancreatic cancer risk in low, intermediate, or high exposure groups by carrying out a meta-analysis, which included 8 studies. But recent findings are somewhat different from the above conclusions. Cui et al^[24]^ showed that pancreatic cancer risk might be reduced by using high-dose aspirin. In 2015, Zhang et al^[25]^ carried out a meta-analysis of 12 studies, which included 8 case–controls and 4 cohorts. They concluded use of aspirin might have a potential to decrease the incidence of pancreatic carcinoma.

Thus, it is essential to further discuss the relationship between use of aspirin and pancreatic cancer risk. In order to study the possibility that aspirin use might decrease the risk of pancreatic cancer, a systematic review and meta-analysis, which contained 12 observational studies was performed.

## 2 Materials and methods

### 2.1 Search strategy

The authors searched Embase, Wangfang (Chinese database) and PubMed databases from inception dates to March 12017. We used the following search terms: (Pancreatic cancer OR Pancreatic Neoplasm OR Pancreatic ductal adenocarcinoma) AND (Aspirin OR ASA). We searched ClinicalTrials.gov for unpublished studies. Moreover, the authors also searched the reference lists of the retrieved articles and it was helpful for finding potential related research.

### 2.2 Study selection

The authors identified studies that met the inclusion criteria:

(1) only case control studies, cohort studies, or RCT are included in the study;
(2) these studies must be related to the study of the relationship between aspirin use and pancreatic cancer risk (including pancreatic cancer incidence or mortality);
(3) an adjusted odds ratio (OR) or relative risk (RR) with 95% confidence intervals (CIs) were provided.

Regarding duplicate publications, the author only selected the most accurate and complete studies.

### 2.3 Study quality assessment

The authenticity and quality of the included studies were assessed by using the Newcastle-Ottawa scale (NOS).^[26]^ The NOS assessment was studied from 3 broad perspectives and got the highest score of 9 points. The risk of the following bias categories was allocated according to the NOS score of each study: low bias risk (7–9 points), moderate risk bias (4–6 points), and high bias risk (<4 points).

The literature search, study selection, and data extraction were conducted independently by 2 investigators (Yanxun Li and Lili Liu). Any disagreements were resolved by discussion between the 2 investigators.

### 2.4 Data extraction

Using standardized data collection form, the following useful data was obtained from all the selected studies: name (together with the first author's name and publication year), study design, study period, study follow-up, study sample size (including both the numbers of cases and controls or the cohort size), study outcomes, the quality score of each study, the ORs, and RRs with corresponding 95% CIs for each category. The supplementary files of each study were also reviewed. Some detailed information was obtained by contacting the authors.

### 2.5 Statistical analysis

In this meta-analysis, the efficiency measure was its related OR and 95% CI. Since the absolute risk of pancreatic cancer is low and OR is mathematically close to the RR in case–control studies, OR was selected to access all the results.^[27]^ Taking the consideration of possible heterogeneity caused by different study designs and different assessments of aspirin use, a random-effect model using the DerSimonian and Kacker method^[28]^ was used to solve above problem. We used the Cochran Q statistic (significance level at *P* < .1) and by estimating *I*^2^ to assessed heterogeneity.^[29]^ Low heterogeneity, moderate heterogeneity, and high heterogeneity were regarded by *I*^2^ statistic.^[29]^ Publication bias was tested by the weighted regression method of Egger et al,^[30]^ and *P* value of <.05 represented statistical significance for publication bias. We used STATA 13.0 (Stata Corp, College Station, TX) to deal with all statistical data.

## 3 Results

### 3.1 Characteristics of selected studies

**Figure 1:**
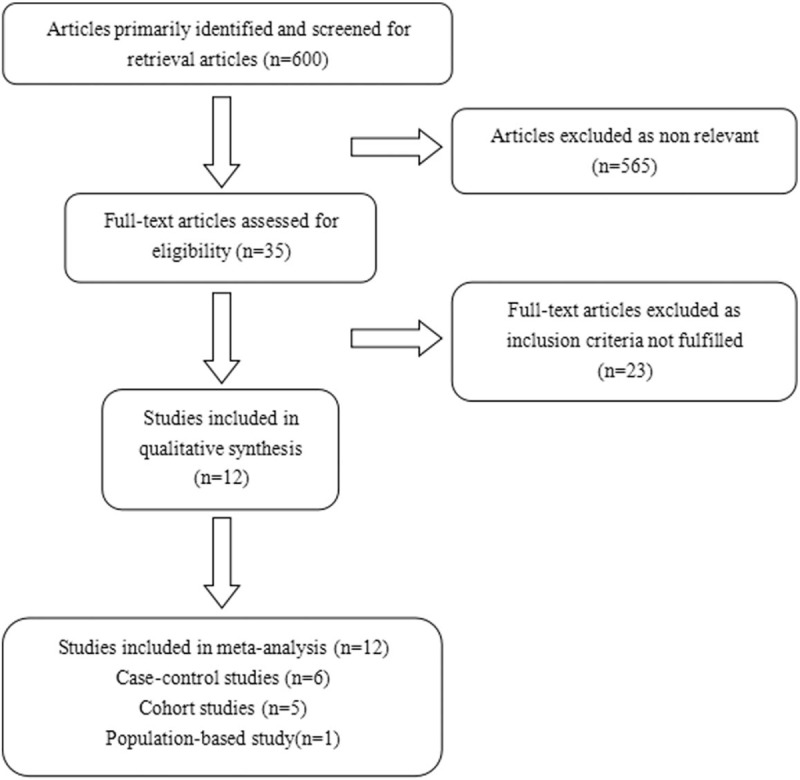
Article selection flow chart.

About 600 potentially publications were yielded in initial search. And then, 565 studies were excluded because their titles and abstracts were not match to the meta-analysis. The full texts of all potentially studies were carefully reviewed, 12 studies were eligible for inclusion in our meta-analysis (see Fig. 1).

The main characteristics of the 12 included studies (6 case–control, 5 cohort studies and 1 population-based study) are shown in Table 1. A total of 12 studies with 4748 pancreatic cancer cases and more than 252,025 healthy controls were available for this meta-analysis. Ten studies addressed use of aspirin in the incidence of pancreatic cancer. We analyzed the relationship between aspirin use and tumor related mortality in 2 cohort studies. The time for the studies was published from 2002 to 2017 year. Of the 12 included studies, eight studies were conducted in the United States, and the participants of the other four studies were Australian, English, Chinese, and Italian. More detailed information is provided in Supplementary Table 2.

**Table 1:**
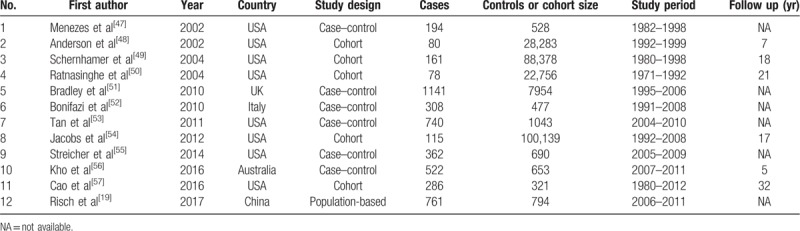
Characteristics of included studies.

### 3.2 Aspirin use and the mortality risk of pancreatic cancer

**Figure 2:**
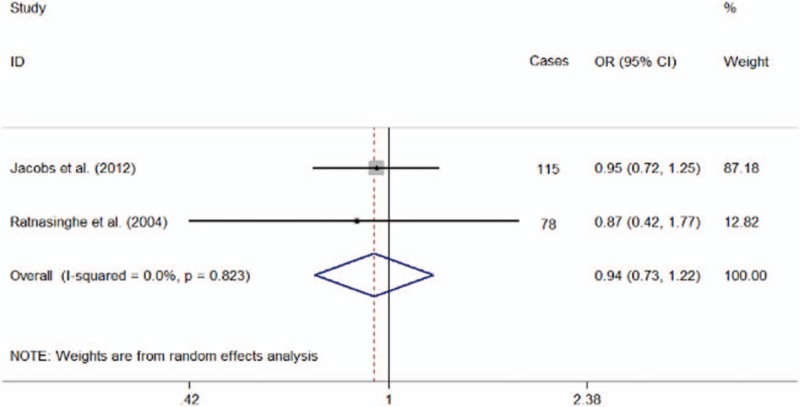
Forest plot showing the association between aspirin use and pancreatic cancer mortality.

A total of 2 studies were enrolled to investigate the association between aspirin use and the mortality risk of pancreatic cancer. Combined analyses inferred that the use of aspirin was not related to the mortality of pancreatic carcinoma (OR = 0.94; 95% CI = 0.73–1.22). Test for heterogeneity showed that there was no statistical significance (*P* = .823; *I*^2^ = 0.0%; Fig. 2).

### 3.3 Aspirin use and the incidence risk of pancreatic cancer

**Figure 3:**
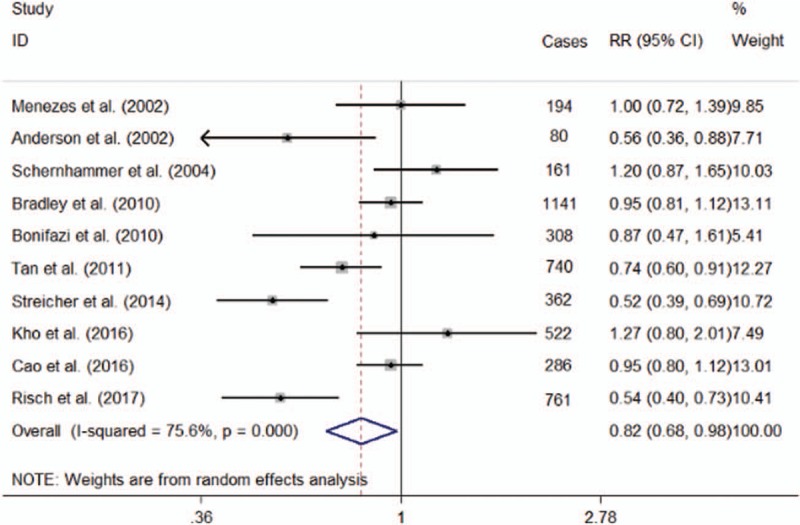
Forest plot showing the association between aspirin use and the incidence of pancreatic cancer.

1. Regular use of aspirin
  This comprehensive analysis contained 10 studies and revealed pancreas-cancer incidence might be decreased by using aspirin (OR = 0.82; 95% CI = 0.68–0.98) with high heterogeneity (*P* = .001; *I*^2^ = 75.6%; Fig. 3). For the cohort studies, the pooled estimate OR was 0.78 (95% CI = 0.55–1.12; *I*^2^ = 83.50%) and 0.84 (95% CI = 0.66–1.06; *I*^2^ = 73.20%) for the case–control studies. Nevertheless, amongst this studies might exist high heterogeneity. Sensitivity analysis showed that eliminating any one of the studies did not substantially change the overall estimate, with an OR range from 0.78 (95% CI = 0.65–0.94) to 0.86 (95% CI = 0.72–1.02). An Egger linear regression test (*P* = .615) did not give us an evidence of publication bias.
2. Subgroups analysis
  (1) the dose of aspirin use
    In our analysis by using incidence as an independent endpoint, we did not find the beneficial effect of large dose of aspirin on pancreas-cancer incidence (OR, 0.95; 95% CI 0.90–1.01; *P* = .098; *I*^2^ = 47.7%). And the same result was seen in the low-dose aspirin intake studies. Above analysis showed that it was no signification association between low-dose aspirin intake and risk for pancreatic cancer (OR, 0.89; 95% CI 0.73–1.07; *P* = .209; *I*^2^ = 76.0%).
  (2) frequency of aspirin use
    The use of aspirin was significantly associated with the prevention of pancreatic cancer. Similar results were found in both high-frequency (OR = 0.67; 95% CI = 0.51–0.87; *P* = .003) and low-frequency (OR = 0.76; 95% CI = 0.62–0.95; *P* = .015) aspirin use. No significant heterogeneity was existed in the both high-frequency (*P* = .117; *I*^2^ = 53.3%) and low-frequency aspirin use studies (*P* = .199; *I*^2^ = 38%).
  (3) duration of aspirin use
    In this meta-analysis, we also studied whether the risk of pancreatic cancer was related with duration of aspirin use. The analysis of 6 studies suggested that if duration of aspirin use was <5 years, it would not decrease the pancreas-cancer incidence (OR = 0.78; 95% CI = 0.59–1.04; *P* = .096), with heterogeneity (*P* = .002; *I*^2^ = 74.2%). In addition, 7 studies revealed that duration more than 5 years was significantly related with a decrease in pancreas-cancer incidence (OR = 0.76; 95% CI = 0.64–0.91; *P* = .003), without obvious heterogeneity among the original studies (*P* = .122; *I*^2^ = 40.4%).

## 4 Discussion

In spite of great progress in diagnosis and treatment, neoplastic disease is still one of the main causes of morbidity and mortality.^[2]^ More and more evidences indicate that aspirin used as an adjuvant therapy for cancer may reduce metastasis and increase the survival rate of cancer patients.^[31]^ In the present study, we indicated that aspirin use may lower pancreatic cancer risk.

In this article we explored whether there is a relationship between the dosage of aspirin and the mortality of pancreatic cancer. However, combined analyses inferred that there was no significant association between aspirin use and pancreatic cancer mortality. The authors known that this discovery should be interpreted cautiously, because a small amount of research is included. However, another study showed that taking aspirin daily for at least 5 years might significantly reduce pancreas-cancer mortality (hazard ratio (HR) = 0.25; 95% CIs = 0.07–0.92).^[31]^

The authors studied the relationship between the use of aspirin and pancreas-cancer incidence. This meta-analysis supported the mechanistic hypothesis that use of aspirin was negatively related to pancreatic cancer risk (OR = 0.82; 95% CI = 0.68–0.98; *P* = .029). The meta-analysis by Zhang et al^[25]^ was found that aspirin use might reduce the incidence of pancreatic cancer (OR = 0.77; 95% CI = 0.62–0.96). This conclusion was consistent with our result. However, the discovery of our meta-analysis is not exactly the same as previous meta-analysis.^[22]^ That article indicated that the use of aspirin has nothing to do with a reduced risk of pancreatic carcinoma.^[22]^ One study^[32]^ found that taking aspirin was related to pancreatic cancer risk. In addition, taking aspirin has shown an inhibitory effect on pancreatic carcinoma in both in vitro and in vivo studies. Our meta-analysis showed that pancreatic cancer incidence might be decreased by using aspirin. However, we realized that a high heterogeneity was existed in our study (*P* = .001; *I*^2^ = 75.6%). It is necessary to carry on sub-analysis.

We studied the potential effects of the dose, frequency and duration of aspirin use. In this article we found that taking aspirin in low dose was not statistically related to reduced risk for pancreatic carcinoma (OR, 0.89; 95% CI = 0.73–1.07; *P* = .209; *I*^2^ = 76.0%). And we also found that using large dose of aspirin might have no effect influence on the incidence of pancreatic cancer (OR, 0.95; 95% CI = 0.90–1.01; *P* = .098; *I*^2^ = 47.7%). One study^[24]^ suggested that high-dose aspirin use might reduce pancreatic cancer risk (OR = 0.88; 95% CI = 0.76–1.01; *P* = .069); when a study^[33]^ that the risk of death was removed from this meta-analysis, the overall risk estimates related with the impact of large-dose aspirin use on cancer risk were significant (OR = 0.78; 95% CI = 0.64–0.95; *P* = .014).^[24]^ Another study^[23]^ did not find chemopreventive effects on pancreatic cancer by using NSAIDs. Our meta-analysis found that not only high-frequency (OR = 0.67; 95% CI = 0.51–0.87; *P* = .003) but also low-frequency aspirin use (OR = 0.76; 95% CI = 0.62–0.95; *P* = .015) might lead to a significant decline in pancreatic cancer incidence. No significant heterogeneity was existed in the both high-frequency (*P* = .117; *I*^2^ = 53.3%) and low-frequency aspirin use studies (*P* = .199; *I*^2^ = 38%). We have not found that continuous use of aspirin has a great risk for the incidence of pancreatic cancer.

We also found that aspirin use duration more than 5 years was significantly related with a decline in the incidence of pancreatic cancer (OR = 0.76; 95% CI = 0.64–0.91; *P* = .003), without obvious heterogeneity among the original studies (*P* = .122; *I*^2^ = 40.4%). Taking aspirin might slow down cancerization rather than prevent initial tumor progression.^[34]^ Rothwell et al^[31]^ revealed that daily use of aspirin might reduce mortality among several common tumors. The lengthening of the duration of treatment increases the benefit.^[31]^ However, 1 study by Cook et al^[35]^ indicated that low-dose aspirin use (75–100 mg) over a 10-year treatment did not lower pancreatic cancer risk.

The molecular mechanisms of aspirin against pancreatic carcinogenesis have been revealed by many experimental researches. Aspirin appears to modulate Wnt signaling at multiple levels including effector pathways of COX-2/PGE2, activity of the β-catenin destruction complex, and the expression of key Wnt target genes involved in tumorigenesis.^[36]^ NO-aspirin showed a good suppression of iNOS, COX-2, and β-catenin protein expressions corresponding to inhibition of pancreatic tumorigenesis.^[37]^ In addition, 1 study suggests that the proliferation of pancreatic cancer cells might be inhibited by taking aspirin and this might be associated with the activity of GSK-3β.^[38]^ Nuclear factor κ B and COX-2 play a critical role in the progress of pancreatic cancer and their biological functions might be suppressed by using aspirin.^[39]^ Taking aspirin may have potential to inhibit the activation of EGFR in pancreatic carcinoma.^[40]^ Cancer cell proliferation might be inhibited by using aspirin which may have potential to modulate the c-MYC oncoprotein.^[41]^ Laboratory studies have shown that aspirin is an ideal candidate for the prevention of tumorigenesis and cannot be ignored in the treatment of malignant tumors.

There are a lot of studies about drug treatment of pancreatic cancer. Combination of gemcitabine and S-1 may be more effective than gemcitabine alone in the treatment of pancreatic cancer.^[42]^ Recently many epidemiological studies have demonstrated the association between aspirin use and cancer risk. A meta-analysis by Tian et al^[43]^ indicated that NSAIDs were significantly related to reduced risk for gastric tumor (OR = 0.81; 95% CI = 0.73–0.89). Rothwell et al^[31]^ reported that taking aspirin could decrease cancer-related death. As the time of treatment was prolonged, the benefit might also increase. Bosetti et al^[44]^ conducted a systematic review, which discussed the association between aspirin use and pancreatic carcinoma risk. However, the study did not suggest the beneficial role of aspirin in risk for pancreatic cancer. In 2014, Wolf et al^[45]^ suggested that there was no correlation between aspirin and the incidence of perioperative bleeding, blood transfusion demand, or the incidence of major postoperative complications. And the study also suggested that daily aspirin therapy was effective and that the continuous use of aspirin should be considered acceptable especially for those who needed antiplatelet therapy. Therefore, the abovementioned published literature might support our findings that aspirin use was related to risk of pancreatic cancer.

Several limitations should be analyzed. The possibility of exposure misclassification for aspirin intake might be one of the limitations. The high and low dose range of aspirin was different in the initial study, because different questionnaires or scales were used to assess aspirin intake. We tried to minimize this inaccuracy by collecting the most similar data across the analysis. Second, this meta-analysis might exist significant heterogeneity. The present existed heterogeneity might be related with geographic area, sex, learning quality, and use-method of aspirin. This may have an impact on our results. In future, we need to conduct a detailed subgroup analysis with stratified the data into subgroups. Third, the possible publication bias might exist in this study. Hopewell et al^[46]^ suggested that the studies with positive results might be relatively easier to be published than negative results. Although it was difficult to exclude all possibility publication bias, no evidence was found in the Egger regression model.

In summary, this meta-analysis suggested that the use of aspirin might be negatively associated with the incidence risk of pancreatic cancer. Specifically, the frequency and duration of aspirin use might play an important role in decreasing the incidence of pancreatic cancer. However, there was no signification association between use of aspirin and mortality risk of pancreatic cancer. Considering the limitations in our study, it is urgent to design high relevant large clinical trials in the future.

## Acknowledgments

Thanks to all investigators who offered help in the systematic meta-analysis.

## Author contributions

**Conceptualization:** Jinjin Sun, Yanxun Li.

**Data curation:** Jinjin Sun, Yanxun Li, Zhijia Jiang.

**Formal analysis:** Jinjin Sun, Yanxun Li, Zhijia Jiang.

**Investigation:** Jinjin Sun, Yanxun Li.

**Methodology:** Jinjin Sun, Yanxun Li.

**Project administration:** Jinjin Sun, Yanxun Li.

**Resources:** Yanxun Li, Lili Liu.

**Software:** Yanxun Li, Lili Liu, Zhijia Jiang.

**Supervision:** Yanxun Li, Lili Liu.

**Validation:** Yanxun Li, Lili Liu.

**Visualization:** Yanxun Li, Lili Liu.

**Writing – original draft:** Yanxun Li, Lili Liu.

**Writing – review & editing:** Yanxun Li, Lili Liu.

## Supplementary Material

Supplemental Digital Content
