# Supplementary material for: Aspirin use and pancreatic cancer risk: A systematic review of observational studies
Source: Medicine (Baltimore). 2019 Dec 20;98(51):e18033. doi: 10.1097/MD.0000000000018033 (PMC6940047; doi:10.1097/MD.0000000000018033)
Supplement: Supplemental Digital Content [file medi-98-e18033-s001.doc]

**Table2. Exposure and Definition of Aspirin Use**

| No. | First Author | Exposure | Definition of  Aspirin Use | Strength of Association  (95% CI) | Confounders For  Adjustment | Score |
| --- | --- | --- | --- | --- | --- | --- |
| 1 | Menezes et al.[47] | aspirin | non-regular used  Dosage of 1–6 tablets/week  Dosage of ≥7 tablets/week  Duration of use for 0.5–10 years  Duration of use for ≥11 years | OR, 1  OR, 1.00(0.72–1.39)  OR, 0.85(0.49–1.45)  OR, 0.82(0.54–1.26)  OR, 1.21(0.81–1.82) | age, sex, race, smoking  status, BMI, family  history of pancreatic  cancer, education | 6^a^ |
| 2 | Anderson et al.[48] | aspirin | never used  ≤ 1 time/week of aspirin  2–5 times/week of aspirin  ≥ 6 times/week of aspirin | RR, 1  RR, 0.75(0.45–1.25)  RR, 0.47(0.22–0.98)  RR, 0.40(0.20–0.82) | age, smoking status,  current multivitamin use, diabetes | 7^a^ |
| 3 | Schernhameretal.[49] | aspirin | non-regular used  current use 1–3 tablets per week  current use 4–6 tablets per week  current use 7–13tablets per week  current use≥14 tablets per week  regular use, 1–5 y  regular use, 6–10 y  regular use, > 10 y | RR, 1  RR, 1.26(0.85–1.85)  RR, 1.41(0.82–2.40)  RR, 1.65(1.05–2.59)  RR, 0.86(0.39–1.89)  RR, 1.12(0.72–1.74)  RR, 1.10(0.64–1.89)  RR, 1.75(1.18–2.60) | age, smoking status,  BMI, diabetes,  non-vigorous physical  activity in metabolic  equivalents per week,  follow-up cycle | 7^a^ |
| 4 | Ratnasinghe et al.[50] | aspirin | no aspirin used  any aspirin used (≥1 times  a week for at least 6months)  use of aspirin( males)  use of aspirin( females) | RR, 1  RR, 0.87(0.42–1.77)  RR, 1.03(0.52–2.07)  RR, 0.67(0.17–2.73) | age, sex, race, smoking  status, BMI, poverty  index, education | 9^a^ |
| 5 | Bradley et al.[51] | aspirin | never used  ever used for aspirin and  derivatives until 1 year before diagnosis  high-dose aspirin(≥300 mg a day) | OR, 1  OR, 0.95(0.81–1.12)  OR, 1.10(0.81–1.50) | smoking status, BMI,  alcohol use, history of  chronic pancreatitis,  history of rheumatoid  arthritis, use of other  drugs, diabetes | 7^a^ |
| 6 | Bonifazi et al.[52] | aspirin | non-regular used (< 1 day/week for more than 6 months)  regular used (≥1 day/week for  more than 6 months)  duration of use < 5 y  duration of use ≥5 y  current users ≥ 5 y | OR, 1  OR, 0.87(0.47–1.61)  OR, 1.40(0.62–3.17)  OR, 0.53(0.21–1.33)  OR, 0.23(0.06–0.90) | age, sex, smoking status, BMI, diabetes, education, study center, year of interview | 8^a^ |
| 7 | Tan et al.[53] | aspirin | never used (< 1day/month)  use 2–5 days per week  use 6+ days per week  aspirin dosage of 1–2 tablets/day  aspirin dosage of 3+ tablets/day | OR, 1  OR, 0.61(0.38–0.96)  OR, 0.63(0.47–0.85)  OR, 0.81(0.63–1.03)  OR, 0.72(0.50–1.04) | age, sex, smoking status, BMI, diabetes | 6^a^ |

| No. | First Author | Exposure | Definition of  Aspirin Use | Strength of Association  (95% CI) | Confounders For  Adjustment | Score |
| --- | --- | --- | --- | --- | --- | --- |
| 8 | Jacobs et al.[54] | aspirin | never used  updated analyses for current  daily use  updated analyses for aspirin  use < 5 y  updated analyses for aspirin  use ≥ 5 y | RR, 1  RR, 0.95(0.72–1.25)  RR, 0.89(0.64–1.23)  RR, 1.03(0.73–1.46) | age, sex, race, smoking  status, BMI, heart  disease, stroke,diabetes, hypertension, cholesterol-lowering  drug use, aspirin use, history of , education | 8^a^ |
| 9 | Streicher et al.[55] | aspirin | never used  regularly used  low-dose (75–325 mg  per day) aspirin  regular-dose  (325–1200 mg every  4 to 6 hours) aspirin  aspirin ≤ 6 y  aspirin > 10 y | OR, 1  OR, 0.52(0.39–0.69)  OR, 0.94(0.91–0.98)  OR, 0.98(0.96–1.01)  OR, 0.50(0.36–0.70)  OR, 0.61(0.37–1.00) | age, sex, race, smoking  status, BMI, diabetes,  blood type, education | 8^a^ |
| 10 | Kho et al.[56] | aspirin | never used  Low-dose aspirin only  Regular-dose aspirin only  Low and regular aspirin  Low-dose < 5 years of use  Low-dose ≥5 years of use | OR, 1  OR,0.81 (0.61–1.08)  OR,1.27 (0.80–2.01)  OR,1.00 (0.52–1.91)  OR,0.76 (0.51–1.12)  OR,0.81 (0.59–1.11) | age, sex, pack-years of smoking, alcohol use,  diabetes history, and adult BMI | 6^a^ |
| 11 | Cao et al.[57] | aspirin | non-regular used  (< 2 times/week)  regularly used  (≥2 times/week 325mg) | RR,1  RR,0.95 (0.80–1.12) | age, sex, cardiac risk factors | 7^a^ |
| 12 | Risch et al.[58] | aspirin | never used  ever used  duration of use < 2 y  duration of use 2–4 y  duration of use ≥ 4 y | OR,1  OR,0.54 (0.40–0.73)  OR,0.69 (0.43–1.10)  OR,0.41 (0.24–0.69)  OR,0.53 (0.33–0.86) | age, sex, smoking  status, BMI, diabetes,  blood type, education, H. pyloriCagA | 7^a^ |

a: quality assessment by Newcastle-Ottawa Scales; CI: confidence interval; OR: odds ratio; RR: relative risk;
